# Advanced ECG Analysis to Evaluate Multimodal Exercise Effects on Cardiovascular Health

**DOI:** 10.3390/medicina61030473

**Published:** 2025-03-07

**Authors:** Ruta Brazdzionyte, Deivydas Velicka, Kristina Motiejunaite, Kristina Poderiene, Zivile Kairiukstiene

**Affiliations:** 1 Institute of Sport Science and Innovations, Lithuanian Sports University, 44221 Kaunas, Lithuania; kristina.motiejunaite@lsu.lt (K.M.); kristina.poderiene@lsu.lt (K.P.); zivile.kairiukstiene@lsu.lt (Z.K.); 2 Department of Sport Medicine, Lithuanian University of Health Sciences, 44307 Kaunas, Lithuania; deivydas.velicka@lsmu.lt

**Keywords:** multimodal exercise program, cardiovascular adaptation, ECG, concatenation, ABP

## Abstract

*Background and Objectives*: Cardiovascular diseases (CVD) are the primary causes of death throughout the world. Engaging in physical activity (PA) is crucial for the prevention of CVD, as a lack of exercise significantly impacts global health. For health promotion purposes, it is important to optimize PA and develop the main physical components. Multimodal exercise program (MEP) interventions cause unique cardiac changes that can be systematically analyzed using advanced ECG techniques. Using algebraic co-integration methods, this study examined the physiological cardiac adaptations of a 6-week MEP compared to sedentary control subjects. *Materials and Methods*: A total of 50 physically inactive males, aged 20–35 years, were recruited for a 6-week MEP. The intervention group (IG) consisted of 28 participants, while the control group (CG) included 22 participants. The MEP included balance, endurance, muscle strength, and flexibility exercises in one session. The cardiovascular system (CVS) was assessed using electrocardiography (ECG) and arterial blood pressure during an incremental cycle ergometer test, both before and after the 6-week period. *Results*: After the post-MEP, the IG’s resting HR showed a slight but insignificant decrease, from 84.5 to 82 bpm, with improved recovery rates at minute 1 (113.1–104.7 bpm). The CG showed a similar trend. pBP in IG significantly increased post-MEP during recovery at minute 1 (73–81) and minute 2 (65–72), where the CG showed a slight but significant difference. DskJT-QRS in IG post-MEP increased significantly during recovery in minutes 1–4, with all values showing *p* < 0.05. CG showed significance only at minute 3. *Conclusions*: Both the 6-week MEP and control had a positive impact on the CVS. The statement refers to changes in dynamic interactions between ECG parameters registered during the incremental exercise test and especially during the recovery after workload. Algebraic data co-integration analysis of ECG parameters demonstrated a sensitive assessment of the influence of exercising on the cardiovascular system.

## 1. Introduction

Cardiovascular diseases (CVD) are the leading causes of death worldwide, responsible for around 20.5 million deaths in 2021, nearly one-third of all deaths globally. The rates of obesity and CVD morbidity are rising, partly due to increasing trends of sedentary lifestyles and physical inactivity, which are significant risk factors for CVD [1]. This rise in CVD-related deaths and morbidity underscores the urgent need for effective prevention and management strategies. To address this growing health issue, promoting PA is crucial. The WHO Global Action Plan on Physical Activity 2018–2030 outlines 20 policy measures aimed at improving PA levels globally [2]. Implementing these measures can help mitigate the risks associated with a sedentary lifestyle and contribute to reducing the burden of CVD by providing numerous cardiovascular benefits through physical activity. This includes decreasing blood pressure, improving insulin sensitivity, achieving a favorable plasma lipoprotein profile, and increasing cardiac output, all of which are supported by evidence from studies on exercise and cardiovascular health [3,4,5]. By prioritizing health promotion and preservation, particularly through increased PA and regular exercise, we can make significant strides in preventing CVD and improving global health outcomes [6].

Routine PA and exercise are crucial for preventing CVD and managing health-related quality of life. Evaluating and scientifically substantiating the effectiveness of different types of PA is a key objective. According to the World Health Organization [7], regular PA of 150–300 min per week, including multi-component exercise involving aerobic activities, systematic strength training, and balance improvement, is a significant factor in well-being and a preventive measure for many chronic diseases.

Multimodal exercise programs (MEPs) that integrate various physical components, such as cardiovascular fitness, muscular strength, balance, and flexibility, into a single session offer a comprehensive approach to enhancing overall fitness. The complementary advantages of MEPs include synergistic effects that improve cardiovascular function through aerobic exercises, which increase cardiac output and enhance vasodilation, while strength training enhances muscle mass and strength, contributing to better metabolic health and reduced CVD risk [8,9,10]. Additionally, flexibility exercises improve range of motion and reduce injury risk, which is crucial for maintaining long-term adherence to exercise programs. Studies by several authors [9,11,12,13] have investigated the effects of MEPs and concluded that these exercises are beneficial for enhancing health, supporting their role in comprehensive fitness programs.

Long-term adaptation to physical exertion involves structural and functional changes in the body that occur over extended periods of consistent training. This adaptation is facilitated by rapid response mechanisms and temporary physiological changes, which allow individuals to handle previously challenging workloads and function efficiently in low-oxygen environments. Research often indicates that significant cardiovascular, skeletal, and nervous system adaptations are typically observed after 12 weeks of training [14,15], although some studies suggest that benefits may also arise within shorter periods, such as 4–8 weeks [16,17]. Essential factors for CVS adaptation include regular PA, dietary changes, elimination of harmful habits, and environmental modifications. While much research has focused on high-performance athletes [18], there is a notable lack of studies examining the functional state of non-athletes who engage in recreational training.

One common form of exercise involves the use of weighted equipment, such a cycle ergometers and treadmills [19,20]. The cycle ergometer test is commonly used for its ability to allow participants to select their workload—whether maximal until voluntary exhaustion, sub-maximal, or dosed—without the anxiety associated with moving surfaces. However, it engages fewer muscle groups compared to treadmill tests. The cycle ergometer is used to assess aerobic capacity through indirect methods, with the functional state evaluated via ECG. This non-invasive and cost-effective tool has been instrumental in identifying cardiac deviations, which is essential for diagnosing CVD [21]. Early detection of at-risk individuals and continuous monitoring of those diagnosed with CVDs are crucial for improving diagnostic and treatment strategies, ultimately reducing premature cardiovascular mortality [22]. The rise in new ECG measuring devices has introduced challenges in data processing and analysis, leading to significant advancements in ECG analysis techniques [23]. The algebraic data cointegration method was developed by scientists to evaluate biological signal analysis. This method allows for assessing the interaction or mutual independence of two signals recorded simultaneously by observing their behavior under the influence of various factors. The recorded ECG parameters (the value of a selected indicator for each cardiac cycle) become a digital time series, and the disc value is calculated between them.

Notably, many recent investigations have proposed and utilized a new method for the evaluation of dynamic interactions between ECG parameters using algebraic co-integration. The most widely investigated inter-parametric concatenations are between the RR and JT intervals and between the RR and QRS intervals [24,25,26].

In this study, we utilized algebraic co-integration to analyze the concatenation of ECG parameters measured at baseline, during a cycle ergometer test, and 5 min post-recovery. We compared the dynamics in inactive participants before and after a 6-week MEP, designed to enhance cardiovascular health and health-related quality of life.

## 2. Materials and Methods

### 2.1. Trial Design

**Figure 1:**
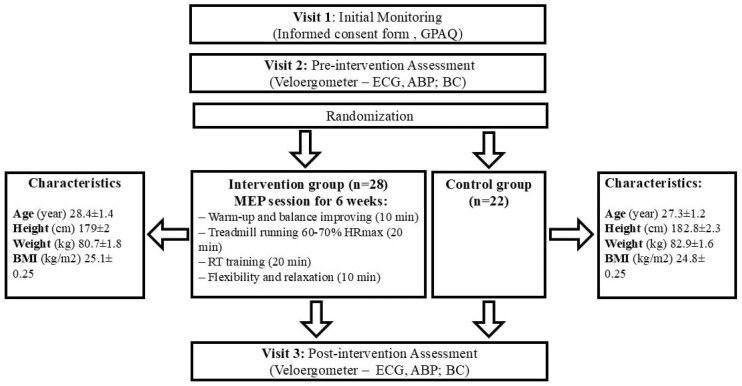
Study protocol. GPAQ: Global Physical Activity Questionnaire; ECG: Electrocardiogram; ABP: Arterial Blood Pressure; BC: Back Check; MEP: Multimodal Exercise Program; BMI: Body Mass Index; RT: Resistance Training.

Before the first measurements were taken, a meeting was organized to inform the participants about the study protocol, including instructions not to smoke or eat for three hours before the examination. All measurements were taken using the same procedure and under the same conditions. Following the initial measurement procedures, participants were divided into two groups: the intervention group (IG), who engaged in MEP for 6 weeks, and the control group (CG), who were asked to maintain their usual daily habits during the 6-week study period. The second measurement procedures were conducted after 6 weeks. Both sessions followed an identical protocol (shown in Figure 1).

Ethical approval for all procedures in this study was obtained from the Kaunas City Regional Biomedical Research Ethics Committee (Approval No. 2020-01-23, Ref. L-20-1/2). The study adhered to the ethical standards and principles of the Declaration of Helsinki.

### 2.2. Participants

Fifty male non-athletes (age 27.8 ± 0.9 years [range 20–35 years]; height 181 ± 1.6 cm; weight 81.8 ± 1.2 kg) who were physically inactive and had not visited a gym in the last three years were recruited through targeted convenience sampling to participate in this study. The criteria for participant selection required that they be physically inactive males between the ages of 20 and 35 years, free from any health issues, normal BP, normal BMI, and not using any drugs or nutritional supplements. All the subjects read and signed the informed consent form with the description of the testing procedures.

### 2.3. Training Intervention

The intervention outlined below took place over a period of 6 weeks and was overseen by certified instructors. To prevent any external factors from affecting the outcomes, participants were advised to maintain their usual daily habits throughout the study. Multimodal training consisted of 10 min of warm-up and balance improvement (single leg stance test), 20 min of treadmill running (70–85% of HR reserve using Polar Vantage-V, Polar Electro, Kempele, Finland), 20 min of resistance training (standard 1RM assessments were conducted using leg press, leg extension, leg flexion, bench press, lat pull-down, abdominal, and back muscles training machines). The load was selected to achieve muscular tonus through strength endurance training, with intensities up to 50% of maximal voluntary contraction. The weight was progressively increased every two weeks by increasing the load based on the standard 1RM, ensuring a structured and consistent intensity progression throughout the six weeks) and 10 min of flexibility (active stretching techniques for muscle groups) and relaxation. The IG committed to attend three training sessions per week for 6 weeks. These were 60 min group sessions supervised by a coach.

### 2.4. Assessments

Strength measurements (Dr. Wolff BackCheck^®^ (Dr. WOLFF Sports & Prevention GmbH, Arnsberg, Germany) maximal isometric force in kilograms, (BC)), patterns of behavior PA (Global Physical Activity Questionnaire (GPAQ)) and rating of perceived exertion (Borg scale) were conducted and changed to endpoints. ECG parameters were measured during the cycle ergometer test at baseline; during light, middle and heavy workloads; and during 5 min of recovery. These parameters were analyzed using the algebraic co-integration method.

#### 2.4.1. Body Composition

Height and bodyweight were measured using calibrated devices while participants were barefoot and wearing only underwear. Height was measured using a SECA^®^ 213 (SECA GmbH & Co, Hamburg, Germany) and bodyweight using a TANITA^®^ BC-545 scale (TANITA Corporation, Tokyo, Japan). Measurements were necessary to determine the strength set points for the Dr. WOLFF BackCheck^®^ 617 and calculate BMI.

#### 2.4.2. Strength

Strength was evaluated using the Dr. WOLFF BackCheck^®^ 617, which measures the isometric force and the balance of forces of separate muscle groups in kilograms. The BC demonstrates high test–retest reliability and criterion validity, making it suitable for scientific research [27,28]. Participants received instructions and were allowed three attempts for each movement, with the best result selected. The selected exercises included: upper body press, upper body pull, core extension, core flexion, left and right lateral core flexion, and left and right hip extension.

#### 2.4.3. Measures of Physical Activity

The assessment of PA patterns was conducted using the WHO Global Physical Activity Questionnaire (GPAQ), which is recognized for its validity and reliability [29]. It aims to assess PA in three key domains: occupational, transport-related, and leisure-time activities. The GPAQ consists of 16 questions designed to capture the intensity, duration and frequency of PA.

#### 2.4.4. ABP Measurements

ABP (Arterial blood pressure) was measured using the cuff method and by listening to the ‘Korotkoff tones’. Pulse blood pressure (pBP) is described as the difference between systolic blood pressure and diastolic blood pressure, which represent the maximal and minimal circulatory pressures during the cardiac cycle, respectively [30].

#### 2.4.5. ECG Measurements

A 12-lead ECG was recorded with the data collected using the CardioScout Multi ECG recorder. The ECG measurements were recorded every 15 s at baseline; during an incremental workload test on a cycle ergometer with the subjects under light, middle, and heavy workloads; and during a 5 min recovery period. Before the test began, the ECG electrodes were applied to the participant’s body, and the participant rested quietly for 30 min to ensure stable baseline readings. Following this, the participant remained seated on the cycle ergometer for an additional 1 min of quiet rest. The cycle ergometer test involved pedaling at a constant rate of 60 rpm, with the workload starting at 50 W. The intensity increased by 50 W every minute until the participant reached a score of nine on the Borg Rating of Perceived Exertion scale and could not continue. Additionally, the workload intensity during the incremental stepwise increase in the workload was controlled by changes in HR. The exercising was stopped while the HR reached or exceeded 85% of the HR reserve. Following the test, the participant rested on the cycle ergometer for 5 min, during which ECG data were continuously recorded for recovery analysis. The analyzed parameters were ABP, heart rate (HR), the JT interval and discriminants (Dsk) between the RR and JT intervals (Dsk_RR-JT_) and between the JT interval and the duration of the QRS complex (Dsk_JT-QRS_).

### 2.5. Data Analysis

#### 2.5.1. Statistical Analysis

SPSS Statistics 26.0 was used for statistical analysis. The results are presented as the arithmetic mean ± standard error. The Kolmogorov–Smirnov test was employed to determine whether the data met the normality assumption. Repeated measures analysis of variance (ANOVA) for dependent samples was used for statistical analysis. *p* < 0.05 was considered to indicate a significant difference.

#### 2.5.2. Mathematical Methods

The statistical approach based on means is insufficient for capturing the synergistic interactions between physiological system parameters and mechanisms during exercise [31]. An algebraic co-integration method has been developed to measure interactions between two physiological parameters [32]. In this method, two synchronized signals are recorded at discrete time intervals and subsequently normalized to the [0; 1] interval. Normalization is crucial for an accurate analysis of interrelations, ensuring that the signals values are adjusted to a common scale. This adjustment uses an equation in which the normalized value is calculated as the difference between the original value and the minimal physiological value, divided by the difference between the maximal and minimal physiological values. These normalized signals are combined into a second-order matrix.

The initial parameters of the matrix, namely the difference (dfr An = xn − yn) and the co-diagonal product (cdp An = ab(xn − 1 − yn − 1)(xn + 1 − yn + 1)) are calculated. Here, xn and yn are real numbers representing recorded parameters. These parameters have more meaningful characteristics, specifically a Dsk.

Dsk An = (dfr An) 2 + 4 cdp An

Larger Dsk values indicate lower-level inter-parametric concatenation, suggesting weak connections between the analyzed parameters. Conversely, small Dsk values that approach zero [32] indicate a substantial interaction between the analyzed parameters. The analyzed values included baseline, the first load at 50 W (min), the average load (selected based on the physical capacity of the subject; middle), the maximum load, which is the last stage depending on the participant’s capacity (max) and a 5 min recovery period. The data are available from the corresponding author upon reasonable request.

## 3. Results

**Figure 2:**
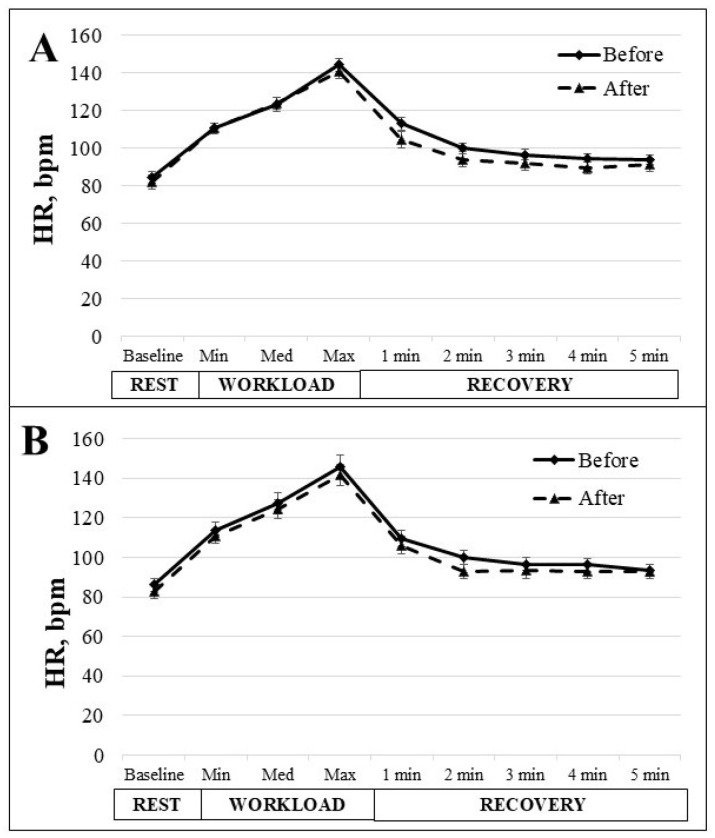
The change in heart rate (HR) in IG (**A**) and CG (**B**) at rest; during the cycle ergometer test with light, middle and heavy workloads; and during 5 min of recovery. The data from before and after the 6-week intervention are shown.

Figure 2 illustrates the change in HR at rest, during the cycle ergometer test, and during recovery for both the IG and the CG. In the IG, the average resting HR was 84.5 ± 3.2 bpm before the 6-week MEP and decreased to 82 ± 3.8 bpm after the MEP. By contrast, the CG exhibited a resting HR of 86 ± 3.4 bpm before the intervention and 82.5 ± 3.4 bpm after, indicating no significant change. During the cycle ergometer test, the HR increased gradually in both groups and peaked during the maximum workload stage. IG reached 144.5 ± 3.5 bpm before the MEP and 140.6 ± 3.6 bpm after the MEP. The CG peak heart rate was 145.6 ± 6.0 bpm before 6 weeks and 141.7 ± 5.5 bpm after. HR was measured every minute for 5 min post-exercise to analyze the recovery phase. The IG showed a faster recovery rate after the 6-week MEP compared to pre-intervention measurements. The IG’s recovery HR in the first minute was 113.1 ± 3.5 bpm before MEP and dropped to 104.7 ± 4.4 bpm after the 6-week MEP. HR gradually decreased in both groups. In the last minute of recovery, HR dropped to 93.8 ± 2.6 bpm before and 91.3 ± 3.5 bpm after the 6-week MEP but did not return to initial values. The CG experienced the same trend in HR changes.

**Figure 3:**
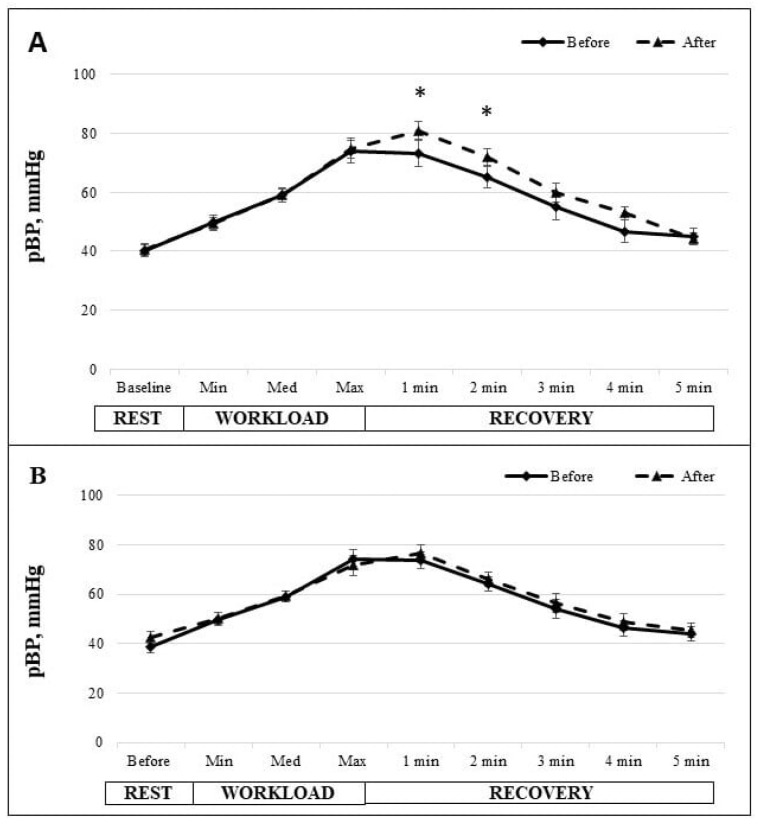
The change in pulse blood pressure (pBP) in IG (**A**) and CG (**B**) at rest; during the cycle ergometer test with light, middle and heavy workloads; and during 5 min of recovery. The data before and after the 6-week intervention are shown. * *p* < 0.05.

Figure 3 presents the dynamics of the pulse blood pressure (pBP) at rest, during the cycle ergometer test, and throughout the recovery period for both the IG and the CG. In the IG, the average resting pBP was recorded at 40 ± 2 mmHg before the 6-week MEP and increased to 41 ± 2.2 mmHg following the program. By contrast, the resting pBP in the CG was 39 ± 1.9 mmHg initially and increased to 43 ± 2.4 mmHg after 6 weeks, indicating no significant change. During the cycle ergometer test, pBP increased progressively in both groups, peaking at maximum workload. The IG peak pBP reached 74 ± 3 mmHg before the MEP and slightly increased to 75 ± 4.2 mmHg after. The CG recorded a peak pBP at 74 ± 3.5 mmHg before and decreased to 72 ± 4.1 mmHg after 6 weeks. The IG presented a significantly higher pBP in the first and second minutes of recovery after the 6-week MEP compared to their initial measurements and the CG measurements. In the first minute of recovery, pBP significantly increased from 73 ± 3.1 mmHg before to 81 ± 4.3 mmHg after the 6-week MEP (*p* < 0.05). The pBP in the second minute of recovery before the 6-week MEP was 65 ± 2.9 mmHg, significantly increasing to 72 ± 3.7 mmHg afterward (*p* < 0.05). Further recovery measurements decreased gradually, but in the last minute of recovery, pBP did not reach the initial values in either group. In the CG, pBP was 45 ± 2 mmHg before the 6-week MEP and decreased to 44 ± 2.9 mmHg after.

**Figure 4:**
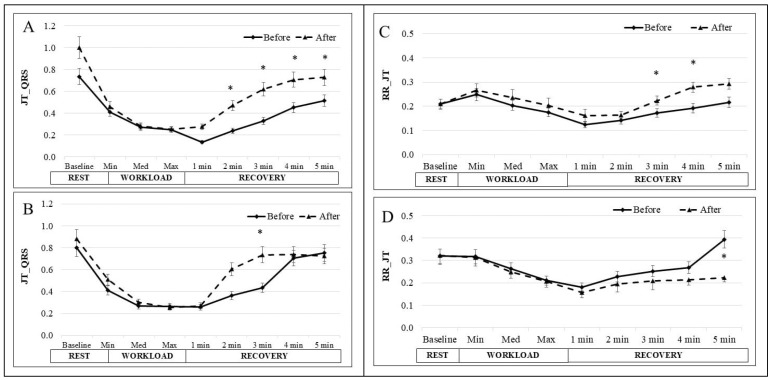
The change in Dsk_JT-QRS_ and Dsk_RR-JT_ in IG (**A,C**) and CG (**B,D**) at rest; during the cycle ergometer test with light, middle and heavy workloads; and during 5 min of recovery. The data before and after the 6-week MEP are shown. * *p* < 0.05.

Figure 4 show the dynamic interaction between the JT interval and the QRS complex (Dsk_JT-QRS_), the RR interval and JT intervals (Dsk_RR-JT_) and the RR interval and QRS complex (Dsk_RR-QRS_) at baseline, during the cycle ergometer test and during recovery before and after the 6-week MEP for the IG and CG. In the IG, the Dsk_JT-QRS_ value at baseline was 0.736 ± 0.1 before the 6-week MEP and 0.917 ± 0.13 after the MEP. In the CG the baseline value before the 6-week MEP was 0.801 ± 0.11 and 0.870 ± 0.15 after. During the workload, Dsk_JT-QRS_ decreased gradually; it was numerically higher after the MEP, but the differences were insignificant. During recovery, Dsk_JT-QRS_ increased but did not reach the baseline value. In the IG, there was a significant difference at minute 1 (0.135 ± 0.03 before, 0.275 ± 0.06 after), minute 2 (0.239 ± 0.04 before, 0.470 ± 0.11 after), minute 3 (0.331 ± 0.04 before, 0.620 ± 0.12 after), and minute 4 (0.452 ± 0.06 before, 0.708 ± 0.12 after) of recovery between before and after the 6-week MEP (*p* < 0.05). The same tendency was observed in the CG, but there was only a significant difference at minute 3 (0.435 ± 0.08 before, 0.736 ± 0.14 after) (*p* < 0.05). The Dsk_RR-JT_ at baseline was 0.209 ± 0.02 before the 6-week MEP and 0.210 ± 0.02 after the MEP in the IG. In the CG, the baseline was 0.319 ± 0.02 initially and 0.322 ± 0.01 after 6 weeks. During the cycle ergometer test, at each workload, Dsk_RR-JT_ was numerically higher after the MEP compared with values before, but the differences were insignificant. During recovery, Dsk_RR-JT_ gradually returned to baseline in both groups but did not reach the initial value. During the dose test, the Dsk_RR-JT_ at minute 3 (0.172 ± 0.02 before and 0.223 ± 0.02 after), at minute 4 (0.192 ± 0.02 before and 0.279 ± 0.02 after), and at minute 5 (0.216 ± 0.021 before and 0.293 ± 0.022 after) of recovery after the intervention were significantly higher than before (*p* < 0.05). In the CG after 6 weeks, there was a significant decrease at minute 5 of recovery from 0.394 ± 0.09 to 0.222 ± 0.02 (*p* < 0.05). There were no significant differences between Dsk_RR-QRS_ in IG and CG.

The isometric force of the different muscle groups increased after the 6-week MEP: upper body press IG +8.2 ± 1.2% and CG +1 ± 0.5%, upper body pull IG +10.4 ± 3.9% and CG −1.3 ± 0.9%, core extension IG +17.4 ± 5.2 * (*p* < 0.05) and CG +0.8 ± 0.9%, core flexion IG +13.4 ± 5.8% * (*p* < 0.05) and CG +2.5 ± 1.9%, left core lateral flexion IG +15.6 ± 4.2 and CG 0%, right core lateral flexion IG +11.7 ± 4.8% and CG 0%, left hip extension IG +10.6 ± 3% and CG +1.6 ± 1.1%, and right hip extension IG +12.9 ± 3.7% and CG +2.6 ± 2.2%. The participants completed the GPAQ, and their responses indicated a low level of PA. During the exercise test, before and after the MEP, participants in IG and CG consistently reported a perceived exertion level of 9 on the Borg Rating of Perceived Exertion scale, indicating a high-intensity effort that was subjectively similar across all participants.

## 4. Discussion

In this study, we aimed to evaluate the effect of an MEP as a strategy for CVD prevention by employing novel algebraic co-integration analysis of electrocardiogram parameters. The application of algebraic analysis to ECG data enables a detailed assessment of the dynamic interactions between various cardiac parameters during exercise interventions. This research focuses on the synergistic effects of diverse exercise modalities, such as strength, endurance, flexibility and balance, on cardiovascular health. Our findings, along with an exploration of underlying mechanisms, seek to provide information for future prevention strategies for CVD. Although the impact of MEP on the cardiovascular system remains underexplored, prior studies have primarily emphasized heart rate variability assessments [33,34]. It was shown that cardiac autonomic recovery occurs more rapidly in individuals with greater aerobic fitness [35]. Nevertheless, current evidence did not allow us to treat the faster recovery after exercising as the most significant factor in reactivating parasympathetic cardiac response. By using algebraic co-integration methods, our study aims to bridge this gap and contribute to a more holistic understanding of the role of MEP in is associated with cardiovascular adaptation.

In this study, MEP was chosen as an intervention to assess CVS adaptation to physical exertion, as it is still unclear which types of PA are most effective for improving overall health and physical fitness. The multimodal exercise approach stands out by targeting multiple fitness components simultaneously, including strength, endurance, flexibility, and balance. While these exercises have gained increasing scientific attention in recent years, they are most studied for their effects on the musculoskeletal, respiratory, and nervous systems, as well as their humoral responses [36]. While these aspects are important, our primary focus was to examine their impact on the CVS, utilizing detailed ECG indicators. Studies like ours are significant not only in the field of sports science but also in practical applications, as they can help to better understand which exercise regimens are most effective in improving cardiovascular health and physical fitness.

The MEP was applied for 6 weeks to induce long-term adaptation, as changes in the body persist for some time after PA, and immediately after exercise, the body must recover before it can replicate the previous load [37]. The duration of recovery can vary from minutes to weeks, depending on the physiological system or the type of physical load [38]. CVS recovery after exercise is a dynamic process with many physiological changes, not merely a return to the pre-exercise state [39]. In this study, we used a 5 min recovery period, but this was insufficient for some cardiovascular parameters to return to their initial levels. Interestingly, significant differences were observed during recovery, but not during the PA itself, highlighting the importance of assessing recovery, as it provides valuable information about an individual’s physical condition.

To perform a deep and comprehensive analysis of the cardiovascular system, we assessed the interaction and dynamics of ABP and ECG parameters during the physical workload sample. The main ECG indicators (HR, JT interval, QRS, ST-depression) and the dynamic interactions between them were analyzed. One of the analyzed indicators was HR, which is used to indirectly measure maximal aerobic power, as it is linearly dependent on the increase in workload and oxygen consumption [40]. The normal resting HR for adults is between 60 and 100 beats per minute [41]. HR was lower during recovery after a 6-week MEP compared to pre-exercise values. A decrease in HR during exercise indicates improved myocardial function [42,43]. Other researchers have also reported that physical training significantly reduces HR after training sessions [44] due to activation of the parasympathetic nervous system after physical exertion [45]. By comparison, while the CD also displayed a similar trend in HR changes during recovery, they did not achieve as significant a reduction in HR, as observed in the IG. Overall, these findings underscore that while both groups improved their post-exercise recovery HR, the intervention group demonstrated a more pronounced enhancement in their recovery speed following their training regimen compared to baseline performance metrics. This suggests that the MEP effectively contributed to better cardiovascular recovery dynamics in participants who engaged in it.

In the CVS evaluation, we also assessed ABP, which was measured during the ergometric test and recovery. The dynamics of the indicators reflected the well-known trend that during physical exertion, sBP increases and dBP decreases [46]. For a more detailed evaluation, we performed pBP assessments. This indicator is the difference between sBP and dBP [47] and is a measure of arterial stiffness, where higher pressure amplitude indicates improved blood flow to the muscle during workload [48]. In our study, pBP was higher during recovery after the 6-week MEP compared to before the MEP, reflecting better interaction between central and peripheral circulation. In the control group, no significant changes were observed after 6 weeks, suggesting that the MEP had a positive impact on circulation during the workload sample.

To obtain a more comprehensive understanding of the functional condition and adaptation of the CVS to MEP, we evaluated the dynamic interactions between ECG parameters. This method offers a more accurate assessment of the CVS, as the human body has numerous regulatory mechanisms (activating and inhibiting) that create synergistic interactions [49]. Conventional ECG evaluation is less sensitive to relatively small changes in functional status, so we applied these methods to evaluate the synergistic properties of the body and its complexity. The concept of matrices used for analyzing relationships between ECG parameters has been presented in the literature [26]. The convergence of various physiological mechanisms is a key feature of body function under intense physical load [50]. Based on this theory, we performed calculations to assess the dynamic interaction between RR and JT intervals, as well as the QRS complex. The algebraic co-integration method reveals the strengthening or weakening of the relationship between the evaluated indicators. It is shown that decreased disc values after loading indicate a strong relationship between the factors influencing the indicator, possibly an intensification of recovery processes. The results obtained in this study—a decrease in the relationship (increase in disc)—indicate that the recovery processes occurred faster. When Dsk values are close to zero, it indicates that these physiological mechanisms or body systems are harmonized at the systemic level [49]. The results showed that Dsk_RR-JT_ and Dsk_JT-QRS_ were stronger during recovery after 6 weeks of MEP compared to the pre-training values, while no significant changes were observed in the CG. These findings illustrate how the body adapts to find an optimal response to increased energy demands. Based on our results and the algebraic method of data integration, we demonstrated that the analysis of complex dynamic systems is a sensitive and informative approach to understanding CVS mechanisms during exercise [51].

This study highlights the effectiveness of a 6-week MEP in improving muscle strength. Despite not receiving a structured resistance intervention, IG participants demonstrated significant increases in core flexion and extension. The IG showed notable improvements across multiple muscle groups: upper body press and pull and hip extension strength, while the CG exhibited minimal changes. While the control group showed improvements, these were generally less pronounced compared to intervention group. These results demonstrate that an MEP effectively enhances muscle strength and functional capacity, supporting its use for overall physical improvement. Our results show that the effect of physical exercises is not limited to the autonomic regulation mechanisms of the heart, but it is likely that both central and peripheral blood flow regulation mechanisms, as well as these structures, are affected. Accordingly, other research methods and new analysis methods were chosen to evaluate the changes occurring.

This study has some limitations that should be considered when interpreting the findings. One notable limitation is the demographic scope, as the study focused only on men aged 20–35. Therefore, the effects of the MEP on cardiovascular health in women and other age groups remain unclear, suggesting a need for further research in more diverse populations. A few methodological factors may also have influenced the results. The relatively small sample size could limit the generalizability of the findings. Additionally, the physical activity of the control group was not objectively monitored, making it difficult to determine its potential impact on the outcomes. Dietary intake was also not controlled, which means that differences in nutrition may have played a role in the results. Finally, the study was not blinded, which could have influenced subjective measures, such as perceived effort. While these factors should be kept in mind, they also highlight areas for improvement in future research and provide useful insights for further studies.

## 5. Conclusions

Both 6-week MEP and control had a positive impact on CVS. The statement is denoted by changes in dynamic interactions between ECG parameters registered during the incremental exercise test, especially during recovery after the workload. Algebraic data co-integration analysis of ECG parameters showed a sensitive assessment of the influence of exercising on the cardiovascular system.

## Data Availability

The data presented in this study are available upon request from the corresponding author.
